# Chronic Diffuse Nephritis

**Published:** 1881-01

**Authors:** 


### II.—A CASE OF CHRONIC DIFFUSE NEPHRITIS.

The previous history is somewhat imperfect, as it was obtained from the coachman. It is as follows: The horse was driven from New York City, on Saturday July 3d, to the town of Roslin, a distance of seventeen miles, and fifteen miles from this place (Huntington). When leaving New York City the animal was in apparent health, but when on the road Saturday, was caught in a heavy shower. On Sunday morning the animal appeared a little unwell, but not enough so to excite any fear on the part of the coachman. The time occupied in driving from Roslin to Huntington was four hours, a slow rate of speed. It may be well to state that the horse was one of a pair, that were attached to a light road wagon, the load two men, so that the work performed by the animal during those two days was of the mildest type. When near Huntington, the horse began to stagger, principally from weakness of the posterior extremities. This was speedily followed by general weakness, and the coachman thought he had an attack of "blind staggers" or vertigo. The coachman said "his left eye went back in his head so far that he thought it would never return." As soon as the horse was stabled, he seemed to partially recover. A few hours after, or at four o'clock P. M., a second attack developed with the following symptoms: Constant pawing with the left forward foot, a constant attempt to rub the loins with his nose, and if let loose upon the open floor would walk round in a ring, always turning to the left. If turned to the right, he would instantly turn to the left when let loose. At nine o'clock P. M. I saw the horse for the first time; he was then standing in his stall, constantly pawing with his left forward foot; was very excitable; eyes prominent and glassy; showed great weakness in his posterior extremities; pulse full and beating 80 to the minute; temperature 104⅕°F.; rectum empty; no apparent desire to micturate, and the coachman informed me that he had not passed water, to his knowledge, since he left New York. The owner informed me that he had always been troubled with his kidneys. I diagnosticated uremic poisoning from renal disease. As the urine could not be obtained, an exact diagnosis of the lesion could not be made. Ordered a brisk cathartic: Aloes Barbadoes 8 drachms, combined with Hydraryg chloride mite 2 drachms, in one ball; took a small quantity of blood. The pulse grew a little stronger; applied warm fomentations to the loins; administered a warm water enema; gave spiritus etheris nitrosi, 2 ounces, in water, as a stimulant and diuretic. The horse died the next morning, at eight o'clock, a little less than twenty-four hours after the exposure to the heavy rain. At the post-mortem, the lungs, heart, liver, spleen, and intestines appeared to be perfectly normal; the kidneys were surrounded by a thick fatty capsule, but the kidney substance was very easily torn or broken when handled. A microscopical examination of the kidneys was made by Drs. Satterthwaite and Porter, at the School of Histology in connection with the Columbia Veterinary College. On dissection, the kidneys were deeply congested. The epithelium in some of the uriniferous tubules was perfectly normal, but in most of the tubules the epithelium was found to be in an advanced state of granular and fatty degeneration. In some of the tubes there were no epithilia, and the tubes were filled with hyaline and fatty masses, probably casts. In some places, where the epithelium was completely destroyed, the walls of the tubes were collapsed and appeared as fibrous cords; there was also some increase in the interstitial tissue. The lesion of the kidney was that of chronic diffuse nephritis, and confirms the diagnosis.

J. Lindsay, D. V. S.,
*Huntington L. I.*

Notes by W. H. Porter.—This case presents some points of interest in comparative medicine. This is still another case in veterinary practice which proves quite conclusively that renal disease is a common disease among horses. It also shows that exposing the animal, and especially the loins, to wet and cold, tends to excite exacerbations, if not to produce renal disease. The microscopical examination showed that the diagnosis had been correctly made, and if the urine had been previously carefully examined, the exact form of kidney lesion might have been predicted. It proves conclusively that a careful chemical and microscopic examination should be made more extensively in veterinary practice. In this case, however, Dr. Lindsay had no chance of procuring any of the urine for examination; but had the horse been seen earlier, and a diagnosis properly made, the life of the animal might have been prolonged.

This case, in connection with two cases reported in the last number of this Journal, by Mr. William Soula, is still stronger proof that partial posterior paralysis is a condition referable to the kidney as the exciting cause.

